# Expression of Syndecan-4 and Correlation with Metastatic Potential in Testicular Germ Cell Tumours

**DOI:** 10.1155/2013/214864

**Published:** 2013-06-15

**Authors:** Vassiliki T. Labropoulou, Spyros S. Skandalis, Panagiota Ravazoula, Petros Perimenis, Nikos K. Karamanos, Haralabos P. Kalofonos, Achilleas D. Theocharis

**Affiliations:** ^1^Hematology Division, Department of Internal Medicine, University Hospital of Patras, 26500 Patras, Greece; ^2^Clinical Oncology Laboratory, Division of Oncology, Department of Medicine, University Hospital of Patras, 26500 Patras, Greece; ^3^Laboratory of Biochemistry, Department of Chemistry, University of Patras, 26500 Patras, Greece; ^4^Department of Pathology, University Hospital of Patras, 26500 Patras, Greece; ^5^Division of Urology, Department of Medicine, University Hospital of Patras, 26500 Patras, Greece

## Abstract

Although syndecan-4 is implicated in cancer progression, there is no information for its role in testicular germ cell tumours (TGCTs). Thus, we examined the expression of syndecan-4 in patients with TGCTs and its correlation with the clinicopathological findings. Immunohistochemical staining in 71 tissue specimens and mRNA analysis revealed significant overexpression of syndecan-4 in TGCTs. In seminomas, high percentage of tumour cells exhibited membranous and/or cytoplasmic staining for syndecan-4 in all cases. Stromal staining for syndecan-4 was found in seminomas and it was associated with nodal metastasis (*P* = 0.04), vascular/lymphatic invasion (*P* = 0.01), and disease stage (*P* = 0.04). Reduced tumour cell associated staining for syndecan-4 was observed in nonseminomatous germ cell tumours (NSGCTs) compared to seminomas. This loss of syndecan-4 was associated with nodal metastasis (*P* = 0.01), vascular/lymphatic invasion (*P* = 0.01), and disease stage (*P* = 0.01). Stromal staining for syndecan-4 in NSGCTs did not correlate with any of the clinicopathological variables. The stromal expression of syndecan-4 in TGCTs was correlated with microvessel density (*P* = 0.03). Our results indicate that syndecan-4 is differentially expressed in seminomas and NSGCTs and might be a useful marker. Stromal staining in seminomas and reduced levels of syndecan-4 in tumour cells in NSGCTs are related to metastatic potential, whereas stromal staining in TGCTs is associated with neovascularization.

## 1. Introduction

Testicular germ cell tumour (TGCT), although relatively rare, is the most common malignancy in men between 15 and 35 years old age group with increasing incidence in the past decades [1, 2]. TGCTs have become one of the most curable solid neoplasms, due to the advantage of diagnostic and therapeutic methods, but still the prognosis of highly advanced cases with bulky metastatic lesions is generally poor. Histologically, the TGCTs can be classified as seminomas germ cell tumours, which originate from undifferentiated germ cells, and nonseminomatous germ cell tumours (NSGCTs), which are arise from undifferentiated (embryonal carcinoma) and differentiated multipotent cells [3]. NSGCTs are generally more aggressive and the histological classification to seminoma or NSGCTs is the most important criterion for the selection of the treatment strategy. In patients with clinical stage I NSGCTs other biological markers apart from the percentage of embryonal carcinoma and the presence of vascular invasion, which are reliable prognostic indicators to identify patients at high risk for occult retroperitoneal disease, have not yet been shown to be of prognostic significance [4]. It has been shown that the presence of vascular invasion is associated with gain of a region at 17q12 and more specifically with the expression of inflammatory cytokine CCL2 in NSGCTs of stage I [5]. We demonstrated recently that the aggressiveness of testicular germ cell tumour cell lines is associated with increased expression of matrix metalloproteinases (MMPs) and reduced expression of tissue inhibitors of matrix metalloproteinases (TIMPs) [6]. Hence it is important to evaluate novel markers for the development and prognosis of TGCTs.

Several studies have already focused on the role of proteoglycans in human tumours [7–11]. Accumulation of versican, an extracellular matrix proteoglycan, has been shown to correlate to the metastatic potential of testicular tumours [12]. Syndecans are integral membrane proteoglycans that are implicated in cell-cell recognition and cell-matrix interactions [11, 13]. Syndecans have a short cytoplasmic domain, one transmembrane, and one extracellular domain. The latter bearing heparan sulphate and or chondroitin sulphate glycosaminoglycan chains are capable of binding various growth factors and matrix molecules [13]. Syndecan-1 is the most thoroughly investigated member of the syndecan family and downregulation of cell membrane syndecan-1 is regarded as initial step towards malignant transformation in various malignancies [11, 13]. Although various studies have focused on the role of other syndecans in malignancies, little is known about the role of syndecan-4 in tumour development. Syndecan-4 mediates breast cancer cell adhesion and spreading [14] but also binds proangiogenic growth factors and cytokines and modulates growth factor/growth factor receptor interactions regulating angiogenic processes [15, 16]. Syndecan-4 potentiates Wnt5a signaling and enhances invasion and metastasis of melanoma cells [17]. The cell surface levels of syndecan-4 are reduced by Wnt5a signaling that promotes its ubiquitination and degradation thus regulating cell adhesion and migration [18]. Syndecan-4 interacts with chemokines through HS chains and promotes tumour cell migration and invasion [19, 20] but also regulates the invasion of K-ras mutant cells in collagen lattice together with integrin *α*2β1 and MT1-MMP [21]. Taken into account the proved role of syndecans in malignancies and the structure/function similarities among syndecans we aimed to study the expression profile of syndecan-4 in TGCTs as well as its association with the metastatic potential of these tumours.

## 2. Material and Methods

### 2.1. Cell Lines and Cultures

The human seminoma cell line JKT-1 was a gift from Patrick Fenichel (University of Nice-Sophia-Antipolis, Faculty of Medicine, Nice, France) [22]. JKT-1 cells were cultured up to 38 passages to avoid the drift of these cells. The molecular signature of JKT-1 cells used in our study was described previously concerning the expression of seminoma markers (placenta alkaline phosphatase, NANOG, OCT3/4, AP2*γ*, and HIWI) [6]. Early passages of JKT-1 cells used in our study express a signature of markers which is still near from the one expressed by seminoma cells allowing their use as a model to study seminomas. Human embryonal carcinoma cell line NTERA-2 and teratocarcinoma cell line NCCIT were purchased from American Type Culture Collection (ATCC, Manassas, VA, USA). The NTERA-2 and JKT-1 cell lines were cultured in DMEM supplemented with 10% fetal calf serum. The NCCIT cell line was cultured in RPMI 1640 supplemented with 10% fetal calf serum. All culture media contained 100 UI/mL penicillin and 100 UI/mL streptomycin. The cell lines were cultured in a humidified atmosphere containing 5% CO_2_ at 37°C.

### 2.2. Patients and Tissue Samples

Primary tumours were obtained at surgery from nine patients with TGCTs (five with seminoma and four with NSGCTs). Six control healthy testicular tissues were taken from autopsies. All tissue samples were frozen immediately and subjected to RNA extraction. 

A retrospective study was performed including 71 patients with TGCTs who had undergone orchiectomy in our hospital. Patients were further treated according to their stage, the histological type, and specific predictive and prognostic factors. Patients with stage I seminoma were treated with 2 cycles of adjuvant chemotherapy based on carboplatin, while patients with stage I NSGCTs were treated with 2–4 cycles of chemotherapy based on bleomycin, etoposide, and carboplatin. Patients with stage II disease were treated with 4 cycles of adjuvant chemotherapy based on bleomycin, etoposide, and carboplatin, while in patients with stage III disease ifosfamide was added in the therapeutic pattern. RPLND was selected for the treatment of patients with NSGCTs with identified residual disease after completion of adjuvant chemotherapy. Tissue samples were selected from the archives of the Pathology Department of the University Hospital of Patras. None of the patients had received prior chemotherapy or irradiation. The median age of the patients at the time of surgery was 30 years, with a range of 17–78 years. All experiments were performed after obtaining informed consent according to the institutional guidelines. Tumour staging and histopathologic findings were assessed according to the American Joint Committee on Cancer. Clinicopathological characteristics of the patients are summarized in Table 1. After an initial review of all available hematoxylin-eosin stained slides of surgical specimens, serial sections from a representative paraffin block of each case were immunostained. The study was performed in accordance with the precepts established by the Helsinki Declaration, approved by Ethic Committee of Patras University Hospital and patients were enrolled after giving written consent. All data were analyzed anonymously.

### 2.3. Immunohistochemistry

Syndecan-4 expression was examined immunohistochemically by using the D-16 goat polyclonal antibody (sc 9499, Santa Cruz, USA) and avidin-biotin-peroxidase complex (Dako Co., Copenhagen, Denmark). Tissue samples were fixed in 10% buffered formalin and embedded in paraffin. Serial 5 *μ*m sections were taken and deparaffinized with xylene and dehydrated with 98% ethanol. Antigen retrieval was performed in a microwave oven in 10 mM citric acid buffer (pH 6.0). Endogenous peroxidase activity was quenched with 3% hydrogen peroxide for 5 min at room temperature. Nonspecific protein binding of the antibodies was blocked by incubation with 3% normal swine serum in PBS for 20 min at room temperature. Slides were incubated with anti-syndecan-4 polyclonal antibody diluted 1 : 150 in PBS containing 1% normal swine serum for 1 hr at room temperature. Obtained antigen-antibody complexes were visualized by 30 min incubation at room temperature, using biotinylated rabbit anti-mouse antibody diluted 1 : 200 and the avidin-biotin-peroxidase technique (Dako Co., Copenhagen, Denmark). The staining was developed with 3,3-diaminobenzidine (DAB)/hydrogen peroxide for 5 min at room temperature and slides were counterstained with hematoxylin. A positive tissue control and a negative reagent control (without primary antibody) were run in parallel. The level of syndecan-4 immunoreactivity in epithelial and stromal cells was expressed by scoring the percentage of syndecan-4 positive cells into three groups: high staining >30% of the cells stained; low staining 10–30% of the cells stained; and negative staining <10% of the cells stained. Syndecan-4 immunoreactivity in the tumour stroma was scored as follows: 0, no staining; 1+, moderate; 2+, strong staining. The level of stromal components immunostaining was graded by scoring the percentage of positivity into two groups: negative (<10% of stromal cells and negative staining of the stroma) and positive (>10% of stromal cells or/and moderate or strong staining of the stroma). Three independent researchers randomly evaluated the specimens using this method.

Endothelial cells in tumour tissues were stained immunohistochemically as described previously [12]. After examination of the slides, six random fields at high magnification (×250) were chosen to be evaluated for the number of microvessels in each slide. The number of microvessels in each section represents the mean of the six independent measurements. The evaluation was performed by three independent investigators blinded to the clinicopathological characteristics and syndecan-4 expression in the corresponding tissues.

### 2.4. Reverse Transcriptase-Polymerase Chain Reaction (RT-PCR) Analysis

Total RNA was isolated from cell cultures and tissues using the NucleoSpin RNA/Protein extraction kit from Macherey-Nagel (MN GmbH & Co., Germany) following DNase treatment to remove DNA contaminations according to the manufacturers' instructions. Total RNA (1 *μ*g) was reverse transcribed using the PrimeScript 1st strand cDNA synthesis kit (Takara Inc.) using random 6 mers primers provided according to standard protocol suggested. For PCR, primers for syndecan-4 “CTCCTAGAAGGCCGATACTTCT and GGACCTCCGTTCTCTCAAAGAT” and the reference gene glyceraldehyde-3-phosphate dehydrogenase (GAPDH) “ACATCATCCCTGCCTCTACTGG and AGTGGGTGTCGCTGTTGAAGTC” were used.

PCR was performed for 35 cycles (initial denaturation at 94°C for 2 min, denaturation at 94°C for 1 min, annealing at 60°C for 1 min, and extension at 72°C for 1 min in each cycle and final extension at 72°C for 10 min) using 50 ng of template according to DyNAzyme II kit (Finnzymes, Finland). PCR products for syndecan-4 and GAPDH were separated by 2% agarose gel electrophoresis and visualized by ethidium bromide staining. The amounts of PCR products were determined by measuring the fluorescence of the bands using UNIDocMV program (UVI Tech). Relative fluorescence for syndecan-4 was obtained by dividing the fluorescence value for syndecan-4 by that of GAPDH.

### 2.5. Statistical Analysis

Data were analyzed using GraphPad Prism (Version 3.0 GraphPad Software Inc., San Diego, CA, USA). Statistical analyses were performed using the Fisher's exact tests to evaluate the associations between clinicopathologic variables and syndecan-4 expression. All tests were two tailed and statistical significance was set at *P* < 0.05. To estimate statistical significance of the differences in RT-PCR analyses as well as of microvessel number with stromal expression of syndecan-4, a two-tailed Student's *t*-test was used.

## 3. Results

### 3.1. RT-PCR Analysis for Syndecan-4 Expression in GCTs

**Figure 1:**
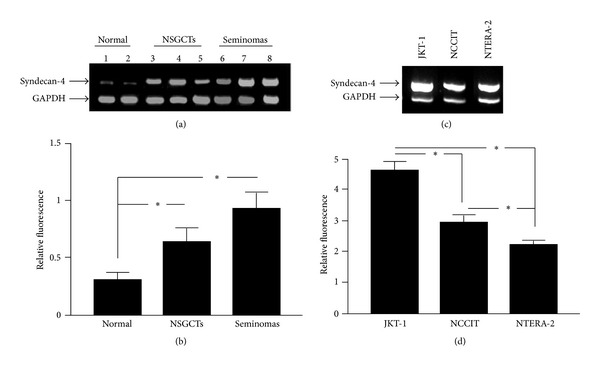
Expression of syndecan-4 in testicular germ cell tumours and cell lines. (a) Indicative RT-PCR analyses of syndecan-4 compared to reference gene GAPDH in two control normal testicular tissues (lanes 1 and 2), three NSGCTs (lanes 3, 4, and 5), and in three seminomas (lanes 6, 7 and 8). (b) Semiquantitative analysis of syndecan-4 expression in normal testicular tissues (*n* = 6), NSGCTs (*n* = 4), and seminomas (*n* = 5). (c) RT-PCR analyses of syndecan-4 compared to GAPDH in testicular GCT cell lines. (d) Semiquantitative analysis of syndecan-4 in TGCT cell lines. The data are presented as the median ± SE and analysed using two-tailed Student's *t*-test (*P < 0.05).

A limited number of tissue samples obtained from patients with TGCTs as well as normal testicular tissues were analyzed for the expression level of syndecan-4 by RT-PCR. As shown in Figures 1(a) and 1(b) low expression for syndecan-4 was found in normal testicular tissues (relative fluorescence median ± SE, 0.30 ± 0.06). Statistically significant increase in the expression for syndecan-4 was detected in both NSGCTs (relative fluorescence median ± SE, 0.64 ± 0.11) and seminomas (relative fluorescence median ± SE, 0.79 ± 0.14) (Figures 1(a) and 1(b)), suggesting a higher expression of syndecan-4 by tumour cells or activated stromal cells. To evaluate the expression levels of syndecan-4 in TGCT cell lines, we performed RT-PCR analysis in three tumour cell lines (Figures 1(c) and 1(d)). Syndecan-4 is highly expressed in seminoma cell line JKT-1 (relative fluorescence median ± SE, 4.64 ± 0.26), whereas statistically significant lower expression was detected in teratocarcinoma cell line NCCIT (relative fluorescence median ± SE, 2.90 ± 0.31) and embryonal carcinoma cell line NTERA-2 (relative fluorescence median ± SE, 2.2 ± 0.19). 

### 3.2. Histological Overview of the Patients

A retrospective study for the expression of syndecan-4 in testicular TGCTs was performed in a population of 71 patients. The histological review (Table 1) of the primary tumours revealed 33 patients (46.5%) with seminoma and 38 patients (53.5%) with NSGCTs. The median age at the time of surgery was 35 years (range 21–78 year) for the patients with seminoma and 26 years (range 17–65) for the patients with NSGCTs. Patients with NSGCTs were divided into three groups: 8 (11.3%) with embryonal carcinoma, 5 (7.0%) with teratoma, and 25 (35.2%) with mixed type TGCTs. Twenty-six of the patients were of *T*_1_ stage, whereas 41 and 4 patients were of *T*_2_ and *T*_3_ stage, respectively. Among the 71 patients with TGCTs, 39 patients (54.9%) were positive for vascular and/or lymphatic invasion and in 35 patients (49.3%) nodal spread of the disease was observed. Only 8 patients (11.3%) were positive for distant metastases (*M*_1_ and *M*_2_). Finally, the categorization of the patients showed that 36 patients (50.7%) were of stage I, whereas 27 (38.0%) and 8 (11.3%) patients were of stage II and stage III, respectively.

### 3.3. Immunohistochemical Expression of Syndecan-4 in GCTs and Correlation with Clinicopathological Variables

**Figure 2:**
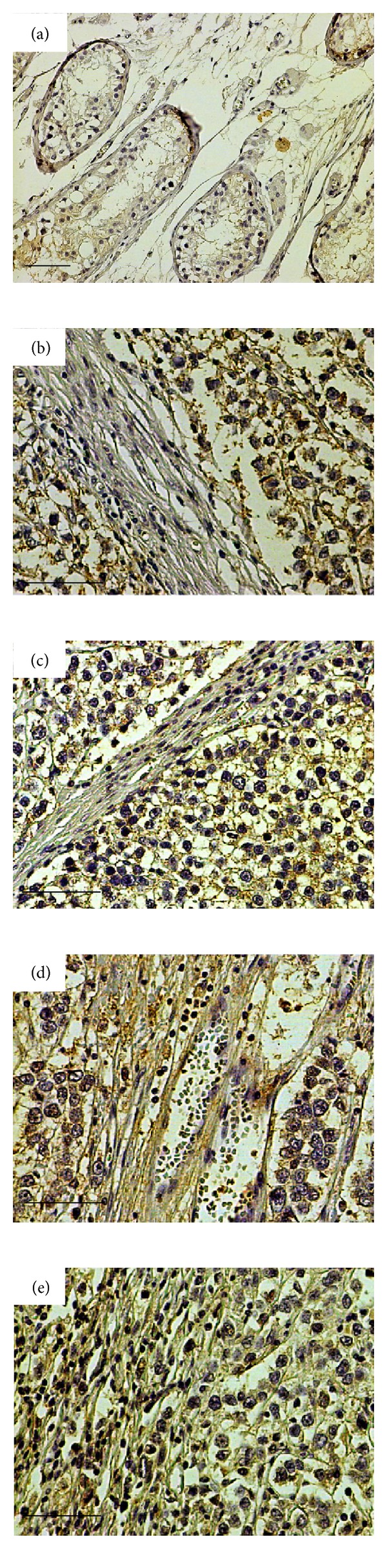
Syndecan-4 is highly expressed in seminomas. Weak staining for syndecan-4 in the seminiferous tubules in normal testicular tissue (a). Tumour cell associated staining for syndecan-4 in stage I seminomas ((b) and (c)) and tumour cell associated and stromal staining for syndecan-4 in stage II seminomas ((d) and (e)). Scale bar denotes 50 *μ*m.

**Figure 3:**
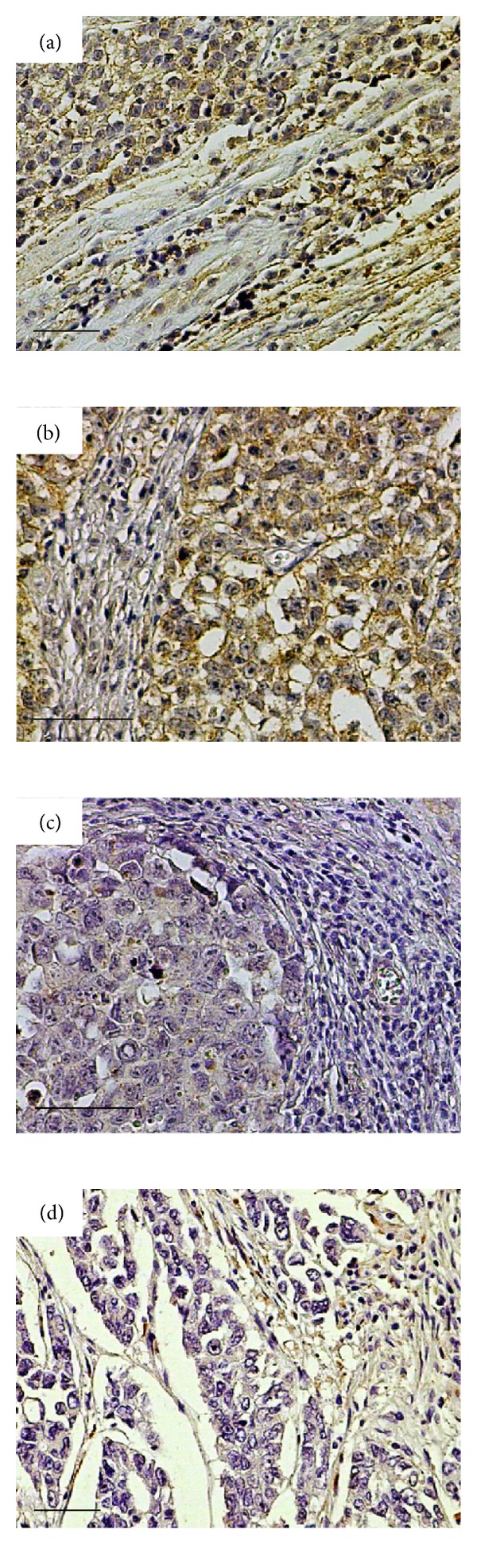
Loss of syndecan-4 staining in aggressive NSGCTs. Tumour cell associated staining and variable stromal staining for syndecan-4 in stage I teratoma/seminoma (a) and stage I embryonal/seminoma (b). Variable stromal staining and loss of tumour cell associated immunoreactivity for syndecan-4 in stage II embryonal/yolk sac (c) and embryonal stage III tumours (d). Scale bar denotes 50 *μ*m.

To evaluate the expression of syndecan-4 by tumour and stromal cells, we performed immunohistochemistry in tissue sections. In normal tests (*n* = 4) weak staining for syndecan-4 was observed in the normal seminiferous tubules showing a cytoplasmic as well as membranous localization with the prominent staining to be detected in the basal cells. No staining was observed in the interstitial connective tissue in the interlobular septa surrounding the seminiferous tubules (Figure 2). Syndecan-4 expression in seminoma (Figures 2(b)–2(e)) and NSGCTs (Figures 3(a)–3(d)) was observed in tumour cells, stromal components, or both. High percentage (>30%) of tumour cells positive for syndecan staining was found in 32/33 (97.0%) patients with seminoma and in 21/38 (55.2%) patients with NSGCTs (Table 2). Stromal syndecan-4 staining was observed in 15/33 (45.5%) patients with seminoma and in 22/38 (57.9%) patients with NSGCTs (Table 2). Syndecan-4 was present in both cell membrane and cytoplasm of tumour cells in seminoma (Figures 2(b)–2(e)) and NSGCTs (Figures 3(a)–3(d)). Stromal syndecan-4 staining was seen both in stromal cells and in collagen tissue mainly in seminomas of advanced stage (Figures 2(d) and 2(e)), and in NSGCTs independently of disease stage (Figure 3). Since high staining for syndecan-4 was observed in tumour cells in all patients with seminoma (Figures 2(b)–2(e)), no correlation with the various clinicopathological variables was demonstrated. In contrast, the stromal expression of syndecan-4 in patients with seminoma was associated with the nodal status (*P* = 0.04), vascular/lymphatic invasion (*P* = 0.01), and stage of disease (*P* = 0.04) (Figures 2(b)–2(e) and Table 3). Stromal syndecan-4 staining was not related to any of the clinicopathologic variable in NSGCTs (Table 4). In NSGCTs less tumour cell associated staining for syndecan-4 was observed in patients with advanced disease stage (Figures 3(c) and 3(d)). In NSGCTs 17/38 patients showed low syndecan-4 expression in contrast to seminoma where 22/23 patients exhibited high syndecan-4 staining (Table 2). This loss of syndecan-4 by tumour cells in NSGCTs was associated with nodal metastasis (*P* = 0.01), vascular and lymphatic invasion (*P* = 0.01), and disease stage (*P* = 0.01) (Figure 3 and Table 4). A clear trend for correlation of lower tumour cells associated staining for syndecan-4 with tumour size and distant metastases was observed as well, but no statistical significance was reached (Table 4).

### 3.4. Correlation between the Stromal Expression of Syndecan-4 and Microvessel Density

**Figure 4:**
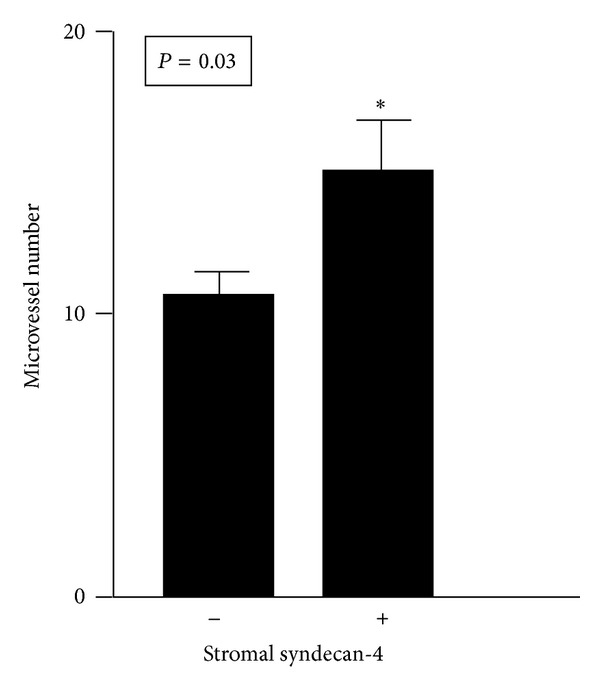
Stromal syndecan-4 promotes angiogenesis. Correlation between stromal syndecan-4 expression and microvessel number in TGCTs. Two-tailed *P* value was obtained by Student's *t*-test.

The expression of syndecan-4 in the tumour stroma was correlated with the microvessel density in TGCTs.  Figure 4 shows that increased staining for syndecan-4 in the tumour stroma was significantly associated with increased microvessel numbers in TGCTs, suggesting an implication in neovascularization.

## 4. Discussion

Syndecans are directly implicated in cancer progression [11, 13]. The aim of the present study was to investigate the expression of syndecan-4 in seminomatous and NSGCTs and to examine all possible associations with the malignant behavior of these tumours. In both seminomatous TGCTs and NSGCTs, significantly increased expression for syndecan-4 was detected in tumour cells. Previously, syndecan-4 has been reported to correlate significantly with high histological grade and negative estrogen receptor status [23], suggesting it to be a marker of poor prognosis in breast cancer. Another study failed to confirm this but instead found syndecan-4 expression to be independent of histological tumour grade and histological tumour type [24]. In our previous study, we demonstrated that estradiol does not affect the levels of syndecan-4 in breast cancer cells through ER*α* signaling although the levels of syndecan-2 are regulated by hormonal treatment [25]. The effects of syndecans in tumour progression may be dependent on organ and tumour type. In this study, the overexpression of syndecan-4 in tumour cells may facilitate the transmission of growth signals in these cells since syndecans are important coreceptors for various growth factors. It has been shown that soluble and membrane-bound forms of syndecan-1 play different roles at different stages of breast cancer progression. The release of soluble syndecan-1 from cell membrane by proteolytic degradation marks a switch from a proliferative to an invasive phenotype in cancer cells [26].

The reduction of syndecans by cancer cell surface is associated with reduced levels of E-cadherin and induction of epithelial to mesenchymal transition (EMT) [26–28]. EMT results in the conversion of malignant epithelial cells into cells with a mesenchymal phenotype and clinically more aggressive tumours. Decreased expression of epithelial syndecan-1 has been reported to be associated with dedifferentiating cancer cells or increasing metastatic potential and to correlate with a poor prognosis in head and neck, gastric, colorectal, laryngeal, cholangiocarcinoma, malignant mesothelioma, hepatocellular, and non-small-cell lung tumours [28–36]. 

Syndecan-4 is a focal adhesion component in a range of cell types, adherent to several different matrix molecules [37, 38], activating protein kinase C-alpha (PKCa), focal adhesion kinase (FAK), and small GTPase Rho to promote cell adhesion and migration [39–46]. FGF-2 treatment of melanoma cells resulted in the reduction in syndecan-4 expression and downregulation of FAK Y397-phosphorylation thus decreasing cell attachment on FN and promoting their migration [47]. Syndecan-4 overexpressing cells form larger and denser focal adhesions, correlated to stronger attachment and decreased cell migration [48], whereas lack of syndecan-4 engagement promotes amotile fibroblast phenotype where FAK and Rho signaling are downregulated and filopodia are extended [49]. These results suggest that a directed homeostasis in syndecan-4 levels supports optimal migration. Our study revealed that seminomatous TGCTs are characterized by much higher staining of syndecan-4 in tumour cells compared to NSGCTs. The lower staining of syndecan-4 in tumour cells is significantly correlate, with nodal metastasis, vascular and lymphatic invasion, and disease stage in NSGCTs. Identical results were obtained by analysis of syndecan-4 expression in TGCT cell lines. Less aggressive seminoma cells JKT-1 [6] exhibited higher expression levels of syndecan-4 more than aggressive NSGCT cell lines such as embryonal carcinoma cell line NTERA-2 and teratocarcinoma cell line NCCIT. Although increased levels of syndecan-4 in tumour cells may promote cell growth, the imbalanced upregulation of syndecan-4 in seminomas may be related to the lower metastatic potential of these cells, which is a general characteristic of this type of testicular tumours. The lower expression of syndecan-4 in NSGCTs compared to seminomas but still higher than that found in the corresponding normal cells is significantly correlated to the metastatic potential of these tumours. These results strengthen the current opinion that the balanced expression of syndecans by tumour cells regulates their spreading. 

Both seminomas and NSGCTs have shown stromal staining for syndecan-4. The presence of syndecan-4 in the tumour stroma was associated with nodal metastasis, vascular and lymphatic invasion, and disease stage only in seminomas. Such stroma immunoreactivity was also reported for syndecan-1 in reactive stromal cells [30, 50, 51]. Since many epithelial mitogens, including FGFs, hepatocyte growth factor (HGF), and heparin-binding epidermal growth factor (HB-EGF), bind to glycosaminoglycan chains of syndecans, it is speculated that syndecans store several growth factors within the tumour stroma and the accumulation of syndecans may contribute to the extensive angiogenesis and stromal proliferation. The expression of syndecans by stromal fibroblasts may create a favorable microenvironment for accelerated tumour cell growth by storing and presenting growth factors to the carcinoma cells. Furthermore, experimental and clinical data have shown that the expression of syndecan-1 by the stromal fibroblasts promotes breast carcinoma growth *in vivo* and stimulates tumour angiogenesis [52, 53]. Our study demonstrates for first time the stromal distribution of syndecan-4 in malignancies. Syndecan-4 present not only in extracellular matrix but also in stromal cells may play a tumour promoting role in TGCTs. Syndecan-4 stromal staining is significantly associated with neovascularization in TGCTs and the metastatic potential only in seminomas and may be involved in the proliferation of reactive stroma, the promotion of angiogenesis, and the formation of chemotactic gradient of growth factors within tumour stroma. In contrast, the stromal expression of syndecan-4 in NSGCTs, which are more aggressive in general, is not important for tumour cells dissemination and this may be only regulated by lower expression of syndecan-4 in tumour cells that directly affects their migratory ability.

## 5. Conclusions

In conclusion, seminomas and NSGCTs are two different categories of testicular tumours with different expression profiles for syndecan-4. Loss of syndecan-4 overexpression on the surface of tumour cells in NSGCTs is correlated with aggressiveness in contrast to less aggressive seminomas where syndecan-4 is highly expressed constantly. Furthermore, stromal expression of syndecan-4 promotes angiogenesis in TGCTs and metastatic potential only in seminomas. Our data suggest that syndecan-4 represents a biological marker in patients with TGCTs and further studies can be performed in order to determine the clinical utility of syndecan-4 expression in predicting occult lymph node disease in patients with stage I NSGCTs. The identification of reliable prognostic risk factors for those patients remains one of the most challenging issues in assigning patients to the best therapeutic options according to their individual risk profiles for metastasis.

## Figures and Tables

**Table 1:** Clinicopathological characteristics of the 71 patients with TGCTs.

Variable	*n*	*% *
Histological type		
Seminoma	33	46.5
Median age: 35 years		
Nonseminoma	38	53.5
Median age: 26 years		
Embryonal carcinoma	8	11.3
Teratoma	5	7.0
Mixed type	25	35.2
Tumour size (*T*)		
*T*_1_	26	36.6
*T*_2_	41	57.7
*T*_3_	4	5.6
Vascular-lymphatic invasion		
Negative	32	45.1
Positive	39	54.9
Nodal status (*N*)		
*N*_0_	36	50.7
*N*_1_	9	12.7
*N*_2_	22	31.0
*N*_3_	4	5.6
Distant metastases (*M*)		
*M*_0_	63	88.7
*M*_1_	7	9.9
*M*_2_	1	1.4
Stage		
I	36	50.7
II	27	38.0
III	8	11.3

**Table 2:** Syndecan-4 expression in 71 patients with testicular tumours.

Histological type	Syndecan-4 positive tumour cells			Syndecan-4 stromal staining	
	<10%	10–30%	>30%	Negative	Positive
Seminoma	0	1	32	18	15
NSGCTs	2	15	21	16	22

**Table 3:** The association between syndecan-4 stromal staining and the clinicopathologic variables of 33 patients with seminoma.

Variable	Negative	Positive	Statistics
Tumour size (*T*)			
*T*_1_	9	6	*P* = 0.73
*T*_2_ + *T*_3_	9	9	
Nodal status (*N*)			
*N*_0_	14	6	*P* = 0.04
*N*_1_ + *N*_2_	4	9	
Vascular-lymphatic invasion			
Negative	15	6	*P* = 0.01
Positive	3	9	
Disease stage			
I	14	6	*P* = 0.04
II	4	9	

**Table 4:** The association between syndecan-4 stromal and tumour cells staining and the clinicopathologic variables of 38 patients with NSGCTs.

Variable	Stromal staining		Statistics	Syndecan-4 positive tumour cells		Statistics
	Negative	Positive		≤30%	>30%	
Tumour size (*T*)						
T_1_	6	4	*P* = 0.27	2	8	*P* = 0.14
T_2_ + T_3_	10	18		15	13	
Nodal status (*N*)						
N_0_	8	8	*P* = 0.51	3	13	*P* = 0.01
N_1_ + N_2_ + N_3_	8	14		14	8	
Distant metastases (*M*)						
M_0_	14	16	*P* = 0.43	11	19	*P* = 0.11
M_1_ + M_2_	2	6		6	2	
Vascular-lymphatic invasion						
Negative	6	5	*P* = 0.47	1	10	*P* = 0.01
Positive	10	17		16	11	
Disease stage						
I	8	8	*P* = 0.51	3	13	*P* = 0.01
II + III	8	14		14	8	
